# A Unique “Composite” PTLD with Diffuse Large B-Cell and T/Anaplastic Large Cell Lymphoma Components Occurring 17 Years after Transplant

**DOI:** 10.1155/2013/386147

**Published:** 2013-05-07

**Authors:** Kristin La Fortune, Dahua Zhang, Gordana Raca, Erik A. Ranheim

**Affiliations:** ^1^Department of Pathology, University of Virginia School of Medicine, Charlottesville, VA 22908, USA; ^2^University of New Mexico School of Medicine, Albuqurque, NM 87131, USA; ^3^Department of Medicine, University of Chicago, Pritzker School of Medicine, Chicago, IL 60637, USA; ^4^Department of Pathology and Laboratory Medicine, University of Wisconsin School of Medicine and Public Health, K4/432 Clinical Science Center, 600 Highland Avenue, Madison, WI 53792, USA

## Abstract

Posttransplant lymphoproliferative disorder (PTLD) comprises a spectrum ranging from polyclonal hyperplasia to aggressive monoclonal lymphomas. The majority of PTLDs are of B-cell origin while T-cell PTLDs and Hodgkin lymphoma-like PTLDs are uncommon. Here, we report a unique case of a 56-year-old man in whom a lymphoma with two distinct components developed as a duodenal mass seventeen years following a combined kidney-pancreas transplant. This PTLD, which has features not previously reported in the literature, consisted of one component of CD20 positive and EBV negative monomorphic diffuse large B-cell lymphoma. The other component showed anaplastic morphology, expressed some but not all T-cell markers, failed to express most B-cell markers except for PAX5, and was diffusely EBV positive. Possible etiologies for this peculiar constellation of findings are discussed and the literature reviewed for “composite-like” lymphomas late in the posttransplant setting.

## 1. Introduction

Post-transplant lymphoproliferative disorder (PTLD) comprises a spectrum of lymphoid proliferations ranging from polyclonal expansions to overt lymphomas [1]. The majority of PTLDs are of B-cell origin and are associated with EBV due to decreased T-cell immune surveillance. T-cell PTLDs are less common, ranging from 4–15% of PTLD cases with an infrequent association with EBV [2]. PTLDs are generally grouped into four categories: (1) early lesions, reactive plasmacytic hyperplasia, or infectious mononucleosis-like, (2) polymorphic PTLD, (3) monomorphic PTLD of B-cell or T-cell type, and (4) Hodgkin's lymphoma (HL) and Hodgkin's lymphoma-like [1]. PTLD with anaplastic large cell lymphoma features is quite rare [3].

In a search of the literature, we found five PTLD cases that involved both B and T lineage either concurrently or successively [4–7]. The interval between organ transplant and development of PTLD ranged from 42 days to 11 years. The involved sites included lymph node, skin, bone marrow, and spleen. In one case, the peripheral blood contained clonal proliferations of both B and T cells after bone marrow transplant [6]. Three cases developed T- and B-PTLD at different sites after either liver or kidney transplant [4, 5, 7]. And finally, a renal transplant recipient developed a cutaneous T-cell PTLD, followed by an EBV-associated PTLD with both B- and T-cell components [8]. 

The current case is unique in its extremely late onset of 17 years and in that two distinct components of lymphoma with different morphology and immunophenotypes coexist in the same isolated location with the conventional diffuse large B-cell lymphoma portion being EBV negative while the anaplastic looking component which expresses T-cells antigens is EBV positive.

## 2. Materials and Methods

### 2.1. Histologic Features and Immunohistochemical Findings

The morphology was assessed on hematoxylin and eosin-stained sections of formalin-fixed, paraffin-embedded tissue. Immunohistochemical studies were performed using an avidin-biotin-peroxidase complex method on an automated immunostainer (Ventana, Tucson, AZ, USA), using antibodies against CD3, CD15, CD30, CD43, CD79a (Ventana), CD5, CD45, ALK-1 (Biocare, Concord, CA, USA), and CD138 (Serotec, Raleigh, NC, USA). Proper positive and negative controls are included. Immunostains for CD2, TIA-1 (T-cell intracellular antigen 1), CD56, CD4, CD7, EBV-LMP, CD8, and PAX5 were performed by USLABS (Irvine, CA, USA). 

### 2.2. In Situ Hybridization

A Ventana automated in situ hybridization instrument was used according to the protocol set by the manufacturer. 

### 2.3. PCR Analysis

Immunoglobulin gene rearrangement PCR and T-cell receptor gene rearrangement PCR were performed on paraffin-embedded tissue at Mayo Medical Laboratories (Rochester, MN, USA). 

### 2.4. Cytogenetic Analysis

Cytogenetic analysis was performed on twenty metaphase cells from overnight and short-term cultures of solid tumor using procedures found in The AGT Cytogenetics Laboratory Manual [9] with modification. 

### 2.5. Fluorescent In Situ Hybridization (FISH)

FISH analysis using DNA probes (LSI BCR/ABL dual color, dual fusion; Vysis-Abbott, Inc., Des Plaines, IL, USA) specific for the ABL gene on 9q34 and the BCR gene on 22q11.2 was performed according to the manufacture's guide on thin paraffin sections. The probes were selected to enumerate chromosomes 9 (ABL probe) and 22 (BCR probe) in the two-cell populations, since near tetraploid karyotype was found in tumor cells by classical cytogenetics.

## 3. Results

### 3.1. Clinical Features

The patient is a 56-year-old male who underwent simultaneous kidney and pancreas transplant in 1989 secondary to diabetes mellitus. His past medical history is otherwise noncontributory. His family history is remarkable for his mother dying of lymphoma at age 48 and his father dying from a primary CNS neoplasm at age 88. His immunosuppression regimen included Azathioprine (75 mg/day), prednisone (7.5 mg/day), and Neoral (cyclosporine, 100 mg/twice per day). He had had no rejection episodes to date with the most recent renal biopsy dated three years prior to lymphoma diagnosis (2006). Four weeks prior to presentation, he had experienced fatigue, malaise, generalized pruritus, anemia, and an unintentional weight loss of 2-3 pounds per week. 

CT scans showed a large apple core lesion measuring 6.3 × 3.9 cm within the duodenum without other significant lymphadenopathy. The patient's past EBV status is not known. Following surgery, the EBV viral load in the peripheral blood was 153 copies/ml. The EBV-VCA (viral capsid antigen) IgM level was 6.37 IU (normal range: 0.00–0.99), and EBV early antigen IgG was 4.72 IU (normal range: 0.00–0.99). EBV-VCA IgG was 8.38 IU, and EBV nuclear antigen IgG was below the limits of detection (performed at ARUP Laboratories, UT, USA). This pattern of the EBV antibodies is consistent with, but not diagnostic of, primary infection, as prior titers for the patient were not available for comparison. 

### 3.2. Pathologic Features

**Figure 1:**
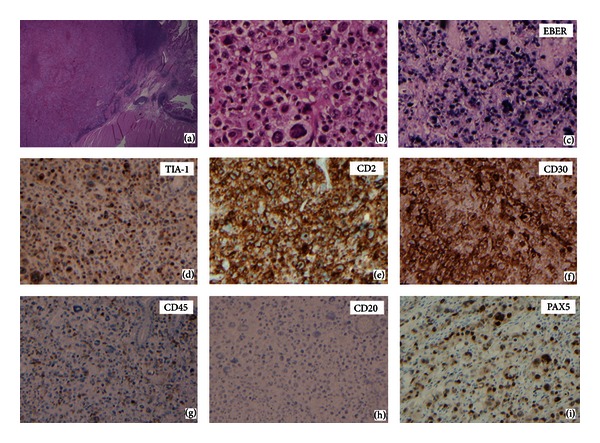
Histopathologic features and immunophenotype of anaplastic appearing area of the lymphoma. (a) The transmural tumor mass in the duodenum with spared small bowel at right (H&E, 20x). (b) Sheets of neoplastic lymphoid infiltrate with anaplastic morphology with horseshoe and ring-shaped multinucleated cells intermixed with other large atypical cells (H&E, 200x). Positive staining for EBER by in situ hybridization (c), TIA-1 (d), CD2 (e), CD30 (f), and variable staining for CD45 wherein most large cells are negative (g). CD20 is absent (h), while PAX5 is present in a nuclear location, as expected (i).

**Figure 2:**
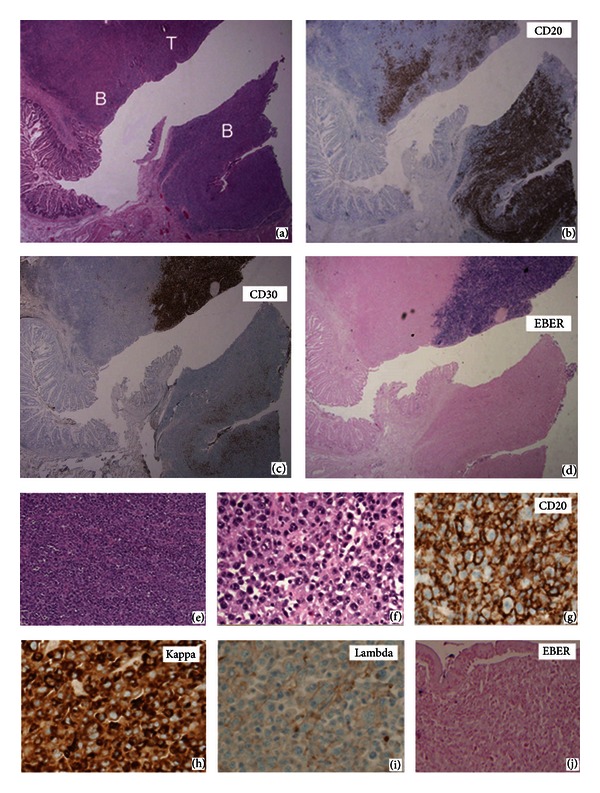
Two distinct components of the lymphoma can be seen in this area of the tumor. (a) H&E section of two distinct components of this lymphoma (20x) with B-cell-like areas marked “B” and T/anaplastic area marked “T.” (b) CD20 stains a distinct area (20x). (c) CD30 positive area is largely CD20 negative (20x). (d) EBER is negative in CD20 positive area (20x). (e) H&E section shows neoplastic lymphoid proliferation in the CD20 positive area (100x). (f) The neoplastic lymphoid cells in CD20 positive area are somewhat less pleomorphic and have fewer multinucleated tumor cells (H&E, 400x). (g) High power view of CD20 positive cells (400x). (h) Positive staining for Kappa light chain (400x). (i) Negative staining for Lambda light chain (400x). (j) Negative EBER by in situ hybridization (400x).

A small bowl resection was performed. Grossly, the 10 cm segment of small bowel contained a luminal circumferential mass measuring 9 × 5 × 2 cm. The surgical margins were grossly uninvolved. The mass was made up of pleomorphic lymphoid cells involving the submucosa, muscularis and extending out to the serosa in places. In some areas, these cells show an anaplastic morphology with horseshoe and ring-shaped multinucleated cells intermixed with other large atypical cells (Figures 1(a) and 1(b)). In other areas, a somewhat more monotonous, immunoblastic, or plasmacytoid appearance is evident (Figures 2(e) and 2(f)).

Immunohistochemical (IHC) staining revealed two distinct populations of tumor cells correlating with their morphologic differences. The first, involving the more anaplastic appearing population, shows expression of CD30 and T-cell antigens TIA-1, CD43, and CD2 and is positive for expression of EBV-RNA (EBER-1) by in situ hybridization (Figure 1) and EBV-LMP (a minority of cells) by IHC (not shown). CD7 is expressed in a minority of the cells in a cytoplasmic and, rarely, membrane pattern. The cells in this component fail to show expression of the T-cell antigens CD3, CD4, CD5, and CD8. CD45 was variably expressed. Intriguingly, these cells show universal nuclear staining for a relatively specific marker of B-cell lineage (see Discussion for exceptions), the transcription factor PAX-5 (Figure 1). The IHC pattern is summarized in Table 1.

The other population of large, immunoblast-like cells shows a more typical B-cell phenotype with expression of CD20, CD79a, and kappa light chain restriction (Figure 2). They also express CD43, which is commonly seen in B-cell lymphomas. These morphologically malignant cells did not express detectable levels of EBER RNA nor EBV LMP-1 protein. Most of these cells appeared to be CD30 negative, though it was difficult to ascertain whether some might express this marker in areas where the two cell types were intermixed. These two-tumor cell populations occupy distinct locations from each other in some areas but are present in a composite fashion overrunning each other in others (Figures 2(a)–2(d)). 

**Figure 3:**
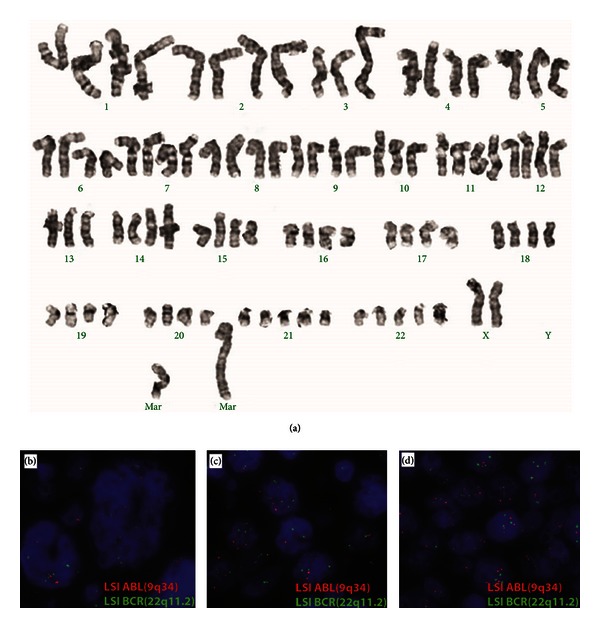
Conventional cytogenetics and FISH analysis of the lymphoma. (a) Representative karyotype showing complex numerical and structural abnormalities. (b) and (c) FISH analysis using LSI ABL (9q34, red) and LSI BCR (22q11.2, green) dual color probes shows approximately 3–8 signals for chromosome 9 (red) and chromosome 22 (green) in anaplastic large cell lymphoma-like area. (d) FISH analysis using the same probes shows approximately 4 signals for chromosome 9 (red) and chromosome 22 (green) in conventional diffuse large B-cell lymphoma area.

Conventional cytogenetic analysis showed a complex karyotype: 84-90,XX,-Y,-Y,-3,t(3;14)(q25;q22)x2, add(3)(q29), add(4)(q31)x2,-5,-6,del(6)(q15q23)x2,-8,-13,-20,+21,+22,+1-2mar. This near-tetraploid abnormal clone (a composite of sixteen cells) contained multiple numeric and complex structural abnormalities including a deletion in the long arm of chromosome 6 from 6q15 to 6q23.  Figure 3(a) shows a karyotype of a representative abnormal cell.

We sought to determine whether the near-tetraploid chromosomal content was present throughout the tumor, or represented a distinct subset arising via fusion of malignant with nonmalignant cells. Fluorescent in situ hybridization study using BCR/ABL probe dual probe as a means to assess ploidy in specific morphologic areas of the tumor showed the majority of cells in diffuse large B-cell lymphoma area to have 4 ABL signals for chromosome 9 (red) and 4 BCR signals for chromosome 22 (green). In the anaplastic area with T- and B-cell antigen expression, the pattern is more complicated; the large horseshoe-shaped cells have more than 4 green and red signals (Figures 3(b) and 3(c)), while the smaller cells have around 4-5 signals for each locus. Even though only 2 chromosomes are analyzed, the pattern suggests that both tumor cell populations share the aneuueploid cytogenetic abnormality but with the very morphologically aberrant cells likely containing even greater genome duplications. Molecular genetic studies found a clonal immunoglobulin rearrangement, but no evidence of clonal T-cell receptor gene rearrangement was detected (performed at Mayo Medical Laboratories).

Staging of the patient, including bone marrow biopsy, confirmed that disease was restricted to the duodenum. Following recovery from surgery, the patient underwent treatment with six cycles of anti-CD20 antibody (Rituxan) plus CHOP chemotherapy. He had no further fevers after initiating chemotherapy and continues to be in clinical and radiographic (PET scan) remission over 6 years following the initiation of chemotherapy.

## 4. Discussion

The case reported here is, to the best of our knowledge, unique in a number of respects. These include the very lengthy period after transplant (17 years) of PTLD occurrence, the location (duodenum), and the apparently dichotomous components harboring EBV in an unexpected pattern, positive in the anaplastic “T-cell-like” cells and negative in the B-cell elements.

The original impressions from H&E sections were of an apparent anaplastic large cell lymphoma with some areas showing somewhat less pleomorphism. In the first block chosen for immunostaining, only a small number of atypical CD20 positive cells were present, while the bulk of the mass as well as focal lymphatic spaces contained CD30^+^ EBER^+^ ALK^−^ CD20^−^ CD79a^−^ CD3^−^ cells. Further staining in an effort to determine lineage on this block further supported T-cell differentiation, with expression of CD43, TIA-1, and CD2 on all the large cells, CD7 expression on a minority, and with absence of the NK marker, CD56, or other T-cell antigens such as CD4 and CD8. Staining for PAX-5, the paired box containing transcription factor also known as BSAP (B-cell-specific activator protein), however, suggested the possibility of B-cell lineage. Normally, PAX-5 is thought to be B cell restricted, marking both B-cell lymphoma and Hodgkin's lymphoma [10], with expression in some AML [11] and neural tumors and carcinomas [12]. It has also been identified in a single T-cell lymphoma [13] and in some T-ALL [14]. Further staining in other areas of the tumor revealed solid areas of CD20^+^CD79a^+^PAX-5^+^CD43^+^ positive cells that lacked T-cell antigens and EBER RNA. Thus the only uniform markers between the two components were CD43 and PAX-5, which stained similarly in each. Clearly, limited diagnostic sampling of the lesion by needle core biopsy or limited pathologic sections could have missed one of the two components.

This case is of interest both as a diagnostic categorization dilemma and a biological puzzle as to how these two distinct tumor cell subsets arose simultaneously in the duodenum seventeen years after transplant. With regard to the latter point, there is a substantial reason to believe that the two biologically distinct components of this tumor are derived from a common precursor rather than representing a true composite lymphoma. First, their appearance at the same, limited anatomic location strongly suggests that the tumor cells are related. Second, despite a preponderance of T-cell antigens in one cell type and B-cell antigens in the other, only immunoglobulin gene analysis showed clonality while T-cell receptor analysis did not. Finally, despite minor variations between different cells within the grossly abnormal cytogenetic analysis, two distinct populations of cells were not apparent. By performing FISH analysis on tissue sections, we were able to ascertain that the roughly tetraploid chromosomal content was present in both the B and T-cell-like areas of the tumor.

In many hematopoietic tumors, the malignant cells show phenotypic heterogeneity, but this usually represents selective loss or gain of one or two normally expressed differentiation antigens or changes associated with differing levels of maturation of tumor cells (e.g., loss of CD20 on some lymphoplasmacytic lymphoma cells as they become “closer” to plasma cell differentiation). In this case, however, only PAX-5, CD43, and variable CD45 expression are shared between the two components, while numerous other relatively T/NK and B-restricted markers differ, as does the presence of EBV. How to explain these findings? One possibility is that a diffuse large B-cell lymphoma was evolving in this patient and a neoplastic cell become EBV infected, changing the cell phenotype dramatically. While aberrant T-cell antigen expression has been associated with EBV infection, this has largely been limited to expression of CD2 and CD3 on a subset of pyothorax-associated lymphomas and on EBV-transformed B-cell lines [15]. The induction of multiple T-cell associated antigens, such as the CD2, CD43, and TIA-1 seen here, simply via EBV infection has not been described, to our knowledge. 

An alternative possibility that we considered was whether a B-cell tumor cell may have fused with an EBV-infected T cell with subsequent silencing of many B-cell markers and maintenance of T-cell antigens. The finding of a near tetraploid chromosomal content on cytogenetic analysis made this theory appealing. However, such a scenario should result in the presence of a clonal TCR gene rearrangement in tumor cells, as the fusing T cell would then be amplified along with the tumor cells, but we failed to find this. In addition, FISH analysis on tissue sections revealed the presence of this near tetraploidy in both the B- and T-cell-like areas of the tumor. These findings argue against a fusion event between a malignant B cell and reactive T cell and favor the evolution of a tumor subclone, perhaps induced by EBV infection, with markedly different phenotypic characteristics from the parent tumor cells.

We categorized this tumor as a monomorphic PTLD, diffuse large B-cell lymphoma (DLBCL) type, with aberrant T-cell antigen expression. As discussed above, we based this on Ig clonality, uniform cytogenetic features throughout the tumor, and the shared expression of PAX-5. The spectrum of findings in this case is unique among reported cases, as best we can discern, either in the post-transplant or nontransplant settings. Diagnostic categories that we considered included a composite lymphoma with DLBCL and ALK negative T/null anaplastic large cell lymphoma (ALCL) or a lymphocyte depleted Hodgkin-like lymphoma.

T cell/null ALCL, particularly associated with EBV expression, is extremely rare in the post-transplant setting, with only a single reported case [3]. Interestingly, this case also was associated with a long post-transplant interval (14 years) and was preceded by a primary EBV infection one year prior to diagnosis. Unlike the current case, this case expressed CD3 and other T-cell antigens, lacked B-cell antigens (PAX-5 was not performed), and showed clonal TCR gene rearrangement.

Hodgkin's lymphoma (HL) and Hodgkin-like entities expressing EBV have been reported following allotransplant [16, 17]. While some of the large cells in the “T cell areas” of the tumor resemble Reed-Sternberg cells, these are admixed with almost no small lymphocytes, atypical even for lymphocyte depleted HL. The large cell phenotype, however, could be compatible with this diagnosis, showing CD30, PAX-5, and variable/weak CD45 expression. A review of classic Hodgkin's lymphoma showed that approximately 5% (12 of 259 cases) expressed at least one T-cell marker in the following order: CD2, CD4, CD3, CD5, and CD8 [18]. Further, 13.7% of stage-IIIB/IV Hodgkin's lymphoma showed expression of TIA-1 in tumor cells [19], despite its supposed T/NK specificity. Kanavaros et al. also reported that two of fifty cases of HL weakly expressed TIA-1 and granzyme B in a proportion of the Reed-Sternberg cells [20]. Although the immunophenotype is compatible with a minority of HL cases, we feel the morphologic findings do not support this diagnosis.

Finally, this case presents some interesting parallels with a rare entity known as pyothorax-associated lymphoma (PAL), a non-Hodgkin's lymphoma of B-cell phenotype developing in the pleural cavity of patients after more than 20-year history of pyothorax resulting from an artificial pneumothorax. It usually shows a diffuse proliferation of large cells of B-cell type with loss of some B-cell markers and gain of aberrant T-cell markers such as CD2, CD3, and CD4 in a proportion of cases. PAL is strongly associated with Epstein-Barr virus (EBV) infection [21, 22]. In contrast to the current case, most PAL cases are reported to exhibit a consistent phenotype throughout the tumor. An interesting exception was reported by Mori et al. [22] in which CD20 and CD3 were coexpressed in most cells but with subsets expressing one or the other. In this case, nearly all cells were positive for EBV. To the best of our knowledge, the patient in this case did not have any long-standing fluid collection either near the tumor site or elsewhere.

In summary, we have reported a unique case of posttransplant lymphoma consisting of two distinct but related cell populations occurring very long after organ transplantation, possibly precipitated by an acute EBV infection. This case emphasizes the need for an extensive tumor sampling and immunohistochemical analysis in atypical cases as our limited initial analysis would have rendered a diagnosis of a T cell/anaplastic PTLD, depriving the patient and clinician of potentially effective therapeutic modalities such as Rituxan. This case also suggests further interesting avenues of investigation of the effect of EBV infection on tumorigenesis and cell phenotype.

## Figures and Tables

**Table 1:** The comparison of immunophenotype between the anaplastic large cell lymphoma-like area and conventional diffuse large  B-cell lymphoma area.

Immunostains	Anaplastic large cell lymphoma-like	Diffuse large B-cell lymphoma area
CD20	−	+
CD79a	−	+
Kappa	−	+
Lambda	−	−
PAX-5	+	+
CD45	Variable, mostly negative	+
CD43	+	+
CD2	+	−
CD3	−	−
CD4	−	−
CD5	−	−
CD7	Some +	−
CD8	−	−
TIA-1	+	−
EBV-EBER	+	−
EBV-LMP	Some +	−
CD30	+	Variable
CD15	−	−
CD138	−	−
ALK	−	−
